# Supplementary material for: Are carbon emissions trading and green financial instruments synergistic? -Comprehensive quantitative research based on content analysis
Source: PLoS One. 2024 Mar 7;19(3):e0298601. doi: 10.1371/journal.pone.0298601 (PMC10919601; doi:10.1371/journal.pone.0298601)
Supplement: S1 Data — (ZIP) [file pone.0298601.s001.zip › Data/地级市Z_F工作报告.zip!/╔┬╬≈/║║╓╨╩╨/└┤╫╘╣1⁄2╓┌║┼í╛┬φ┐╦╩2╛▌═°í┐_2008.docx]

各位代表：

　　现在，我代表市人民政府向大会作工作报告，请予审议，并请市政协委员和其他列席人员提出意见。

　　一、2007年工作回顾

　　过去的一年，在市委的正确领导下，全市上下以邓小平理论和“三个代表”重要思想为指导，深入贯彻落实科学发展观，克难奋进，扎实工作，圆满完成了市三届人大二次会议确定的目标任务。实现生产总值291.21亿元，增长12.8%；完成固定资产投资120.05亿元，增长32.1%；实现财政总收入24.0亿元，增长21.7%，其中地方财政收入达到10.23亿元，增长33.0%；城镇居民人均可支配收入8209元，农民人均纯收入2393元，分别增长18.5%和17.4%；万元生产总值综合能耗下降4.5%。

　　——项目建设取得丰硕成果。55个重点建设项目完成投资46.2亿元，建成项目30个；40个重大前期项目取得突破。城褒一级公路建成通车，宁棋高速公路建设顺利。卡房水库基本建成，千山、沙河水库除险加固工程即将验收。大唐略电一期技改、勉县330KV等3个输变电工程建成投用，开工建设水电站26座，建成7座。全年签订经济协作项目266个，总投资141.39亿元，资金到位28.67亿元，增长12.5%。

　　——工业经济增势强劲。规模以上工业完成产值239.49亿元，实现利税25.9亿元，分别增长20.9%和47.6%，产销率达95.3%。18户市属企业完成改制任务，18个重点工业技改项目完成投资13.88亿元，汉川机床和汉江机床扩能改造、八一锌业综合回收、青啤10万吨啤酒扩产等项目相继建成，创省级名牌产品24个。汉中工业技术研究院挂牌成立。外贸进出口2944万美元，增长38.2%。

　　——城市功能大幅提升。完成中心城区风貌特色、西汉三遗址等专项规划编制。收储出让土地1476亩，收益2.34亿元；充分发挥城投公司融资平台作用，争取开发银行贷款4.25亿元。西排洪渠污水截流干管、益州路、南团结街、南郑大道西段、北一环中段等工程相继建成，滨江河堤、中心广场、万邦广场等工程建设接近尾声。城市管理体制理顺，管理水平明显提高。

　　——旅游等第三产业快速发展。全年接待海外游客1.1万人次，国内游客732万人次，旅游总收入17.2亿元，增长36.5%。完成粮食流通体制改革阶段性任务。“万村千乡”和“双百市场”建设扎实推进。全市社会消费品零售总额87.0亿元，增长18.6%。金融机构年末各项存款余额净增34.63亿元，贷款余额净增13.84亿元。

　　——新农村建设步伐加快。建成通村水泥路1431公里，户用沼气池2.02万口，移民搬迁1.3万人，解决了17.97万农村人口的饮水安全和0.85万人用电问题，5.01万人解决温饱，7.78万人实现脱贫。全年粮食总产121.8万吨，油料总产15.2万吨，生猪饲养量534.7万头，茶叶总产0.84万吨，柑桔19.6万吨，蔬菜132万吨，水产品产量2万吨。经过整合的“汉中仙毫”茶叶获得国家地理标志产品保护，新增农业产业化龙头企业25家。劳务输出63.9万人，收入40多亿元。

　　——和谐汉中建设稳步推进。完成财政支出48.4亿元，增长48.4%，关系群众生活与社会稳定的重点支出和公共支出得到有效保障。以“两免一补”为主的教育投入政策全面落实，“两基”工作通过国家复验。完成了45个乡镇卫生院项目建设，城市社区卫生覆盖率达87.5%。“55315”科技创新工程实施顺利，完成专利登记202件。人口自然增长率稳定在2.46‰。文化体育事业进一步发展，农村广播电视“村村通”建设成效显著。新增就业岗位3.6万个，城镇登记失业率控制在3.9%。7.4万城镇居民和25.6万农村贫困人口纳入低保救助，有9.6万人次贫困家庭的患病群众得到医疗救助。植树造林31.5万亩。主要污染物二氧化硫、化学需氧量分别削减8.2%和3.66%。妥善处置了“3.15”特大道路交通事故等突发事件和其他不稳定因素，社会政治安定。以防汛救灾为主的自然灾害防御工作取得全面胜利。信访工作“一控双降”目标顺利实现。公众治安满意率列全省第一。荣获全国双拥模范城称号。

　　——政府承诺的“十件大事”取得实效。西汉高速公路如期全线贯通；机场建设方案得到省委、省政府和空军认可；六大企业集团拉动规模以上工业增长7.9个百分点；农业“四大工程”全面完成年度任务；汉江桥闸建成并下闸蓄水，“一江两岸”景观核心区初步形成；创建中国优秀旅游城市提前一年达标，创建省级卫生城市进展顺利；中小学D级危房全部拆除，138所危改学校建成投用；农村新合疗提前一年实现11县区全覆盖，参合率达89.8%；落实计生奖扶资金1535.7万元；“两见警”等警务创新稳步推进，建成2840个农村警务室和128个社区警务室。

　　——政府自身建设得到加强。全面推行依法行政和行政执法责任制，“五五”普法深入开展，依法治市成效显著。自觉接受人大、政协监督，建议、提案办结率100%，满意率98%以上。受理回复市长热线、市长信箱群众来电来函2309件，建立政府信息公开制度，我市获全国政务公开先进单位。效能监察试点和治理商业贿赂取得成效。进一步完善目标责任考核体系，督查督办、考评奖惩的工作推进机制基本形成。人事、审计、统计、人防、外侨、质监、民族、宗教、对台、档案、地震、气象、慈善、地方志、预备役和残疾人等各项工作取得新成绩。

　　在肯定成绩的同时，我们也清醒地看到经济社会发展依然面临的严峻挑战和存在的突出问题。一是国家实行从紧的货币政策和严格的土地政策，对经济增长依赖投资拉动带来很大压力；二是GDP增速低于全省平均水平1.6个百分点，与兄弟市的差距仍在拉大，实现在陕南率先突破发展任重道远；三是增长方式比较粗放，节能减排任务十分艰巨；四是重大项目特别是重大产业项目缺乏，招商引资机制不活，全民创业氛围不浓；五是城乡居民收入与全省平均水平差距较大，消费价格指数居高不下；六是思想解放和开拓创新的意识不强，一些干部谋大事、干大事的胆略和气魄不够，干事创业的冲劲不足。对此，我们将高度重视，采取有效措施，下功夫予以解决。

　　各位代表，过去的一年是我市经济快速增长的一年，也是综合实力、发展活力和对外影响力不断增强的一年。全市人均生产总值突破1000美元，汉中发展进入新的阶段，站在了新的历史起点。这些成绩的取得，是省委、省政府和市委正确领导的结果，是市人大、市政协监督支持的结果，是全市人民辛勤努力的结果。在此，我谨代表市人民政府，向奋战在全市各条战线的工人、农民、知识分子、干部、驻汉部队指战员、武警官兵和公安干警致以崇高的敬意！向大力支持政府工作的各位代表、各位委员和社会各界人士表示衷心的感谢！

　　二、今年经济社会发展的总体要求

　　2008年是贯彻落实党的十七大精神，实现率先突破发展的重要一年。按照市委三届三次全会的总体部署，今年政府工作的总体要求是：高举中国特色社会主义伟大旗帜，以邓小平理论和“三个代表”重要思想为指导，深入贯彻落实科学发展观，全面贯彻省委十一届二次全会和市委三届三次全会精神，坚定不移地实施“开放兴市、工业强市、农业稳市、旅游活市”和“发展绿色产业、建设绿色汉中”战略，掀起新一轮思想解放，积极推进改革开放和自主创新，着力优化经济结构和转变发展方式，扎实推进新农村建设和基础设施建设，切实加强节能减排和生态环境保护，努力保障和改善民生，不断加强政府自身建设，开拓创新，真抓实干，奋力开创率先突破发展新局面。

　　主要目标是：生产总值增长12.5%；全社会固定资产投资完成145亿元以上；地方财政收入增长20.2%；单位生产总值能耗降低4.2%，二氧化硫、化学需氧量分别削减4.17%和1.65%；社会消费品零售总额增长15%；城镇新增就业2.4万人，城镇登记失业率控制在4.6%左右；城镇居民人均可支配收入和农民人均纯收入分别增长11%和9.1%；居民消费价格总水平涨幅控制在5%以内；人口自然增长率控制在4‰以内。

　　完成上述目标，要着力把握好以下五点：

　　更加注重以思想大解放增强创新活力。以更大的魄力解放思想、更新观念，下功夫改变传统思维定势，打破僵化体制机制束缚，坚持用创新的思路谋求发展，用改革的办法解决发展中的问题，用市场的手段调节经济，用统筹的方法指导工作。

　　更加注重以加快发展保证率先突破。发展相对滞后是我们的基本市情，加快发展是我市率先突破的首要任务。必须在科学发展观统领下，按照又好又快的要求，挖掘更大的发展潜力，谋求更快的发展速度，逐步缩小与发达地区的差距。

更加注重以项目建设推进投资拉动。把项目作为率先突破的重中之重，积极谋划和争取一批事关全局的重大项目，论证和储备一批带动作用强的新项目，推介和招引一批延伸产业链条的好项目，用项目支撑发展，用项目带动发展，用项目引领发展。

　　更加注重以结构调整转变发展方式。围绕做大做强装备制造、有色冶金、绿色产业、生态旅游四大产业，不断优化产业结构、产品结构、企业组织结构和资本结构，促进现代工业、现代农业、现代服务业加快发展，提升经济增长的质量和效益。

　　更加注重以关注民生构建社会和谐。把实施好民生八大工程作为对执政能力、为民理念、工作作风的检验和考验，坚持从群众最关心的事情抓起，举全市之力，切实把省委、省政府部署的民生八大工程落到实处。通过增加城乡居民收入普惠民生，发展社会公共事业服务民生，构建社会和谐保障民生，让人民群众共享改革发展成果。

　　三、今年经济社会发展的重点

　　（一）以做大做强企业集团为重点，加快推进新型工业化

　　大力推进工业结构调整。进一步加强六大企业集团建设，加大汉江机床、汉江药业等8户明星企业培育力度。推动驻汉企业与地方经济融合，促进军工企业加快转型。抓好汉中航空工业园和铺镇、褒河机械加工等7个工业园区建设。整合矿产资源，扶持优势企业的联合重组。确保规模以上工业总产值增长不低于20%。

　　加快国企改革和企业技改步伐。抓好海红轴承厂等8户企业政策性破产和汉中合成化工厂等5户企业的改制，完成汉变公司与西电公司重组。建成汉中卷烟厂新生产线、陕飞公司飞机转包生产条件等4个产业化项目，加快八一锌业年产20万吨锌冶炼、汉中数控机床基地等17个技改项目建设。

　　为企业提供优质服务。加强煤电油运协调，有效解决工业发展中存在的突出问题。做好中航电测、汉川机床等企业上市准备工作，确保今年有1户企业进入资本市场融资。强化以质取胜、品牌带动，扩大省级名牌、著名商标和国家免检产品数量。

　　（二）以加快农业产业化为重点，扎实推进新农村建设

　　加快现代农业发展。稳定粮油种植面积，粮油总产分别达到110万吨、15万吨以上。大力发展“一村一品”、“一县一业”，积极实施60万亩高产密植生态茶园和60万亩优质柑桔现代农业产业化项目，新建无性系良种茶园1万亩，低改5万亩，加快汉中茶城建设；新建和低改桔园各5万亩。大力发展规模养殖场，生猪饲养量力争达到550万头以上；加快现代中药产业发展；新发展设施蔬菜2万亩；积极发展渔业和特种养殖。强化农业科技支撑，推广优良品种，建立健全农业科技创新服务体系和动植物疫病防控体系，积极发展农民专业合作社和农村服务组织，推进订单生产，培育龙头企业，加快农业产业化进程。

扎实推进新农村建设。进一步加大新农村规划和基础设施建设力度，新建1200公里通村水泥路，农村户用沼气池1.5万口，移民搬迁1.1万人。编制完成25个建制镇总体规划和500个村庄建设规划。加快“十镇百村”基础设施项目建设，搞好高速公路沿线2000户民房整治改造。统筹建设农村社会事业，启动建设扶贫重点村150个。突出抓好农业基础设施建设，加大农业综合开发力度，加强农田水利和以天然林保护、退耕还林、水土保持和水源地建设为重点的生态项目建设，完成各类造林25万亩。

千方百计增加农民收入。进一步落实好各项支农惠农政策，稳定完善农村基本经营制度。不断深化农村改革，探索土地流转和规模经营方式。大力发展乡镇企业，促进农民就业、创业和增收。加强进城务工农民的技能培训、权益维护与组织化输出，全年输出65万人，劳务收入力争达到45亿元。

　　（三）以增强城市功能为重点，不断提高城镇化水平

　　高起点规划城市。强化城市规划刚性管理，严格依法办事。切实抓好城镇和风景名胜区规划管理，提高城市控制性详规编制覆盖率，完成中心城区专项规划编制，推进风貌特色规划的应用。抓紧做好高速公路沿线城镇建设规划。

高标准建设城市。坚持城市经营、多元投入的市场化融资方式，实施城建项目33个。建成南郑大道东段、滨江景观休闲带、完成拜将坛扩建、将坛东路风貌街区改造等10个项目；加快中心城区排污主干管等5个项目建设；开工建设汉中城区东、北两个出入口和梁州路南段等12个项目；积极做好汉江新桥、城区集中供热等6个项目前期工作。加快公厕、垃圾站点、集贸市场、停车场等市政公用设施建设。办好中国历史文化名城西北片区年会。实施城乡建设用地存量挖潜，依法清理闲置土地。

高效能管理城市。持续开展专项整治，加大脏乱差治理力度，对城区街道实行分类管理，规范各类市场和摊点。加快绿地建设，规范无障碍设施管理。加强社区服务体系建设，完善城市功能，提升城市品位。建成省级卫生城市，启动创建省级园林城市和文明城市。

　　（四）以综合发挥旅游业功能为重点，加快现代服务业发展

　　加快旅游产品开发。精心打造长青华阳、黎坪景区，争取年内全面推向市场，勉县三国文化旅游区建成国家4A级景区，南湖—红寺湖、张良庙—紫柏山等重点景区建设有突破性进展。发挥好旅游发展基金的“杠杆”作用，吸引社会资本投向旅游开发，集中力量打造精品景区。全面推进旅游景区经营权与所有权分离。支持有条件的县创建省级旅游强县。

　　提升旅游服务水平。深入开展“迎奥运.旅游服务质量提升年”活动，推进行业标准化管理。加快市、县旅游咨询服务中心和星级宾馆、经济型酒店建设。促进文化与旅游的融合，加强非物质文化遗产保护，抓好张骞墓丝绸之路世界文化遗产申报工作。力争全年接待海外游客1.3万人次，国内游客850万人次，旅游总收入23亿元。

　　强化旅游宣传促销。主动适应假日制度改革的旅游发展趋势，以关中和成渝两个客源市场为重点，创新宣传形式和手段，加强区域协作，加大宣传推介力度。开发特色旅游产品，延伸产业链，提高附加值。集中搞好两个大型节庆。鼓励国内有实力的旅行社和中介机构整体策划、代理汉中旅游线路产品。

　　着力发展现代服务业。加快发展物流业、连锁经营等现代流通组织形式，引导城乡居民扩大对信息、保险、教育、旅游娱乐等消费需求。大力培育汉中特色餐饮品牌。发挥供销社农村网点优势，加快“万村千乡”、“双百市场”等新农村流通网络建设。

　　（五）以扩大招商引资为重点，强力推进项目建设

　　努力扩大招商引资。完善招商引资优惠政策和考核体系，明确县区和政府部门年度招商引资硬指标。以珠三角、长三角等发达地区为重点，有选择和针对性地承接产业转移。全年招商引资增长20%以上。加快建设汉中口岸，搭建对外贸易平台，自营进出口总额增长12%。

　　千方百计争取项目。建立项目工作联动机制，完善充实市县两级项目库，加快项目前期工作步伐。以只争朝夕的精神，谋大项目、想大项目、干大项目，紧跟产业政策争跑项目，围绕资源和市场诚招项目，实现项目工作新突破。

　　切实优化投资环境。完善和强化招商引资服务中心功能，加强跟踪服务。实行“一个项目、一个领导、一套班子、一笔经费、一抓到底”的工作机制。实行投资环境评价制度，健全投诉受理机制，严肃查处破坏投资环境的人和事。

　　集中抓好重点项目建设。今年安排重点建设项目55个，年度计划投资50.2亿元；重大前期项目36个。建成宁棋高速公路，开工建设十天高速公路西乡试验段，完成两条国省道改造和384.2公里通乡油路、县乡公路建设；进一步加强与空军、西部机场集团的沟通，机场建设前期工作力争有新的突破。加快汉江桥闸回水段堤防工程建设，实施红花寺等9座病险水库除险加固工程，抓好水库移民后期扶持工作。开工建设330KV洋县、略阳等输变电工程，抓好嘉陵江干流梯级电站开发和小水电建设。

　　（六）以提高财政保障能力为重点，推动县域经济更快发展

　　努力做好财税金融工作。坚持依法治税，优化税收服务，提高财政收入质量。健全财政收支激励约束机制，加强预算管理与审计监督，积极探索财政支出绩效评价机制。大力推动金融生态建设，积极创新金融服务，扩大开发性融资，促进重点企业与金融机构建立战略合作关系。深入推进城乡信用社改革，支持地方金融机构加快发展。加强监管，防范和化解金融风险。

　　加快发展县域经济。以县城和小城镇为依托，以优势产业为载体，以骨干企业为支撑，形成各具特色的发展格局，实现县区优势互补、相互促进、竞相发展。扩权强县试点县和省对县财政管理体制改革试点县要积极争取省上重点支持。

　　大力发展非公有制经济。认真贯彻中、省有关加快发展非公有制经济的政策措施，降低市场准入门槛，激活民间资本进入法律法规未禁止的一切行业和领域。加快县区中小企业融资担保体系建设，着力解决贷款难问题。力争非公经济增加值增长16%。

　　（七）以构建和谐汉中为重点，统筹发展社会各项事业

　　坚持教育科技优先发展。完善城乡义务教育保障机制，提高农村义务教育公用经费补助标准，搞好素质教育，规范民办教育，大力发展职业教育。积极实施“双高普九”。加快汉中职业技术学院发展，尽快开工建设新校区。加快推进科技创新工程，切实保障科技投入，促进科技成果转化。

　　加快发展卫生、文化等各项社会事业。加快县级医疗机构、乡镇卫生院、社区卫生服务中心基础设施建设。完善公共卫生和医疗服务体系，逐步提高城市社区公共卫生服务补助标准和农村新合疗保障水平。加强人口和计划生育工作，稳定低生育水平，改善人口结构，提高人口素质。加强“两馆一站”等基层文化设施建设，打造地方舞台艺术精品，推动文化大发展大繁荣。广泛开展“全民健身与奥运同行”活动，加快体育运动中心建设。建成市广电中心大楼，积极推进农村广播电视“村村通”建设。切实做好民族、宗教工作。

　　加快社会保障体系建设。全面落实就业再就业扶持政策，加强就业指导，重点解决“零就业”家庭就业问题。依法规范企业用工行为，切实维护劳动者合法权益。完善社会保障体系，逐步扩大养老、医疗、失业、工伤、生育保险覆盖范围。完善社会救助体系，规范城乡医疗救助制度。加快住房保障制度建设，开工建设20万平方米经济适用房和2万平方米廉租住房。加强市场监管和价格执法，控制物价过快上涨，保障市场供应，确保低收入居民生活水平不降低。

　　加强资源能源节约和环境保护。实行节能减排工作责任制和问责制，控制新增排污量，关闭大唐略电4号、5号发电机组和两个小水泥厂。落实新上项目环评制度，建立矿山生态恢复治理保证金制度。确保市污水处理厂现有设施正常运行，加快建设污水处理厂二期工程、危险废物集中处置中心，开工建设江南污水处理厂。加强汉江水源地保护，积极争取中省生态补偿和财政转移支付扶持。加强城市环境污染整治，中心城区空气质量二级和好于二级的天数达到310天以上，饮用水源水质合格率达到100%。

　　高度重视安全生产。严格安全责任追究制度，认真落实“隐患治理年”各项要求，强化对道路交通、煤矿、民爆物品、学校、特种设备、建筑施工等重点行业和领域的专项整治，努力消除各种隐患。强化食品药品安全监管。加强突发公共事件应急体系建设。

　　深化“平安汉中”建设。延伸“两见警”和“三深入”工作，深化社区农村警务，确保“三基”建设任务全面完成。开展“农村平安年”建设活动，推进群防群治。继续抓好“五五”普法，推进依法治市。落实信访责任，完善矛盾纠纷排查化解机制，从源头上减少和解决信访问题。

　　（八）以实施民生八大工程为重点，实现发展成果人民共享

　　民生连着民心，民心关乎和谐。今年市政府将把实施民生八大工程作为重中之重，纳入目标责任考核，并在此基础上全力办好八件实事：一是新建改建校舍100所6.28万平方米。二是新型农村合作医疗人员的补助标准从40元提高到80元，新农合参合率不低于85%；社区卫生服务中心覆盖率达80%以上。三是实施农村安居工程，消除全市2255户茅草房。四是建成人饮工程400处，解决18万农村人口的安全饮水问题。五是全面实施农村信息进村入户工程，新建800个农村信息服务站。六是提高城乡低保差额补助标准，基本实现低保对象“应保尽保”和“按标施保”。七是下岗失业人员再就业0.8万人，培训农村劳动力30万人，通过创业带动2万人就业。八是新建和改建3个县级敬老院、7个乡镇区域性敬老院，新增供养床位2250张，五保集中供养率达到20%以上。

　　四、切实加强政府自身建设

　　面对新形势、新任务，我们要以更加开阔的视野、更加饱满的热情、更加扎实的工作，切实加强政府自身建设。

　　科学民主行政。切实加强政府工作人员特别是领导干部的学习教育，全面把握科学发展观的科学内涵和精神实质，牢固树立忧患意识和强烈的发展意识、开放意识、创新意识，科学分析判断形势、把握规律、研究重大问题，进一步增强工作的前瞻性、预见性和主动性。健全重大事项集体决策、专家咨询、社会公示与听证等制度，推动决策科学化、民主化。

　　服务为民行政。牢固树立“全体部门都是服务部门，全体岗位都是服务岗位，全体公务员都是服务员”的理念，将关注民生、服务发展作为政府工作的出发点和落脚点，进一步创新工作方式，简化办事程序，加快办事节奏，提高办事效率，努力为人民群众、企业发展提供更为便捷、经济和有效的服务。

　　依法规范行政。认真落实行政执法责任制，努力做到程序合法、高效便民、权责统一。突出服务事项、服务承诺、收费依据、办事时限、责任追究等关键环节，深入推进政务公开。主动接受人大、政协监督，充分发挥社会监督、舆论监督和政府内部监督的作用，保证公共权力在法律和政策范围内阳光运行。

　　廉洁自律行政。落实廉政建设责任制，完善惩治和预防腐败体系，努力从源头上预防和治理腐败。大力纠正部门和行业的不正之风，深入开展教育收费、医疗服务、社保基金等领域的监督检查。加强效能监察，严肃查处行政“不作为”、“慢作为”、“乱作为”。全体政府工作人员任何时候都要树立正确的权力观、地位观，常修为政之德，常存敬畏之念，常思贪欲之害，常怀律己之心，坚持堂堂正正做人，踏踏实实做事，清清白白行政，真正做到权为民所用，情为民所系，利为民所谋，以勤政廉洁的实际行动，赢得全市人民的信赖和支持。

　　高效务实行政。实现今年的目标关键在谋划，要害在落实。要进一步完善目标责任考核，对重大决策、重要部署、重点项目的完成情况进行跟踪督查和综合考评，促进各级政府工作人员把精力集中在想干事上，把本领体现在会干事上，把目标锁定在干成事上。努力做到定下来的事雷厉风行、抓紧实施，部署了的工作一抓到底、抓出成效。把想干的事情看准，把看准的事情干成，把干成的事情做精。大力推进责任政府、法治政府、廉洁政府、诚信政府建设，不断增强政府的凝聚力、公信力、执行力和推动力。

　　各位代表！汉中是一方充满希望和活力的热土，正焕发着前所未有的勃勃生机。让我们在市委的领导下，团结依靠全市人民，以高昂的斗志锐意改革创新，以饱满的热情全力狠抓落实，以务实的作风推动率先发展，同心同德，埋头苦干，为开创汉中率先突破发展新局面而努力奋斗！
